# Data-driven forwarding: a typology of digital platforms for road freight transport management

**DOI:** 10.1007/s12525-022-00540-4

**Published:** 2022-04-18

**Authors:** Christoph Heinbach, Jan Beinke, Friedemann Kammler, Oliver Thomas

**Affiliations:** 1 German Research Center for Artificial Intelligence, Smart Enterprise Engineering, Parkstr. 40, 49080 Osnabrück, Germany; 2 Osnabrück University, Information Management and Information Systems, Parkstr. 40, 49080 Osnabrück, Germany

**Keywords:** Digital platform, Transport management, End-to-end visibility, Freight forwarding, Data-driven service systems, Smart logistics, O32

## Abstract

The omnipresence of digital platforms (DPs) across industries yields platform-based business concepts that disrupt the road freight market, enabling the digitalization of road freight transport management (RFTM). However, the data-driven service capabilities of DPs in supporting RFTM are manifold, and platform research provides opportunities to explore the emerging digital business concepts following the core process of transport management systems (TMSs). This, in particular, results from the side of road freight operators engaged in the transport management process that are increasingly forced to provide customer-centric RFTM via DPs to remain profitable and competitive within a fragmented business environment. Against this backdrop, this paper aims to explore DPs in the road freight transport domain to gain insights into digital freight services and support logistics companies involved in the transportation process with a novel navigation for the identification of required platform-based services. Following the grounded theory methodology, we present a morphological box encompassing 14 dimensions and eight DP types aligned to RFTM. We reveal digital services of DP visibility, optimization, and analytics. With the insights obtained by our research, we contribute to developing a comprehensive understanding of DPs for the enhanced decision-making of transport stakeholders in the area of digital transport management. Our findings provide an established theoretical research ground that guides platforms as markets for practitioners and proposes further research avenues for scholars toward data-driven and digitalized transport logistics.

## Introduction

“The reality is that the traditional freight forwarder model remains surprisingly analogue, using systems and processes that are slow and inefficient, with opaque pricing and limited use of technology.” (Fraser Robinson, Beacon co-founder and CEO)^1^

In a data-driven world, the omnipresence of digital platforms (DPs) enables transactions for products and services between demand and supply sides (i.e., price matching for consumers and product suggestions or the recommendation of transaction partners) (Armstrong, 2006; Rochet & Tirole, 2003; Tiwana, 2013). Since transport logistics relies significantly on innovative information systems (IS) (Bilyalova et al., 2021), DPs emerge particularly in the European logistics sector, which represents a vital backbone for economies, with an annual turnover rate of 1120 billion EUR (Schwemmer, 2019, p. 7). However, the use of platform technologies has a tradition in the road freight market and is motivated economically. For instance, several decades ago, the electronic marketplace Timocom started a platform business to matchmake freight and truck load capacities between road carriers, freight forwarders, and shippers with the aim of orchestrating freight resources in a fragmented market and exchanging shipment data between shippers and carriers (Lin et al., 2002; Zhang et al., 2008). Currently, digital transport services using cloud infrastructure are being developed particularly by start- ups with high financial volumes that transform the road freight business industry as emphasized by the consultancy company Oliver Wyman (2020). Transport customers use the service offerings of emerging DPs in addition to their transport management systems (TMSs) to enhance the order flow, support operational decision-making, increase visibility, and automate processes (Aviles & Rutner, 2012). Although DPs appear in various forms in the road freight market to leverage digital transformation, a concise distinction of their digital capabilities and service concepts in connection with TMSs has not yet been introduced in scientific discussions.

Scholarly discussions on DP theory have gained insights on economic and engineering perspectives and drawn a line of interaction for technological platforms. “The economics perspective focuses on how platforms as markets mediate transactions across different customer groups and how network effects fuel platform competition” (Gawer, 2014, p. 1240). Given the number of transport stakeholders in a dynamic road freight market utilizing siloed IS for forwarding operations (Heinbach et al., 2021a), the rise of DPs in the forwarding business is primarily linked to technological platforms as markets that promote competition separately from cloud architectures to innovate products and design platforms. More specifically, we have noted that digital freight services based on cloud infrastructures facilitate interactions among several participants (Evans & Gawer, 2016). Thus, in our study, we consider DPs in the context of freight technologies that are “divided into three different types (Evans & Gawer, 2016): transaction platforms (e.g., Timocom), data-focusing platforms (e.g., Cargonexx), and integration platforms (e.g., RIO Cloud)” (Heinbach et al., 2021b, p. 614). These platform concepts serve the same road freight transport market and have recently been explored by scholars through the lens of digitalization. For example, Hofmann and Osterwalder (2017) investigate platform business types in logistics and differentiate between logistics marketplace platforms and cargo space platforms from a multisided perspective (Hagiu & Wright, 2015). In addition, the authors identify vehicle manufacturing platforms that offer data-mining services based on telematics for fleet management following a product-as-a-platform concept. Meanwhile, Elbert and Gleser (2019) shed light on an emerging platform-based logistics business concept named “digital forwarder,” which utilizes DPs to offer electronic freight forwarding services in conformity with the legal character of traditional freight forwarders incorporating transport liability. This new concept has particularly received attention in practice since competitive digital road freight services are developed along the shipment lifecycle in addition to the services offered by traditional freight forwarders leading to substitutional market effects due to their IT competencies and scalable business models (Ortwein & Kuchinke, 2021). However, the vast number of existing platform market terms and concepts indicates the complexity of the dynamic freight forwarding industry and calls for a clear differentiation for both scholars and practitioners to navigate in the sphere of digital transport management. That is, for instance, buying customers of transport services with digital service requirements (e.g., order transmission, electronic invoicing) to be served by road freight carriers or forwarders involving a suitable group of DPs to meet these requirements. As a result, the identified DP group will promote competitive business by specifying providers to be considered by carriers or forwarders for the actual scope of service agreement with customers. In view of the above, a scientific approach to differentiate real-world DPs will help to guide transport stakeholders exploring platform-based business concepts with relevance to an uncovered digital value in logistics fostering platform competition.

Digital services provided by DPs utilize technology to facilitate business processes (De Reuver et al., 2018). Consequently, increasing platform business enables freight forwarding services that follow established business process operations in practice, discussed as road freight transport management (RFTM). In this light, the process of RFTM provides an overarching structure to assign digital freight services offered by DPs and understand aligned platform capabilities from a user perspective. Additionally, this approach emerges particularly from the side of traditional software vendors for TMSs, such as SAP or L-Base, which shift their applications from on- premise to cloud infrastructure. In essence, TMSs address the core business activities for freight transportation on a shipment level, while DPs enrich the operational processes particularly for inbound and outbound logistics (Zutshi & Grilo, 2019) to digitize business transactions (Hofmann & Osterwalder, 2017). Thus, traditional TMS vendors increasingly compete with burgeoning DPs that aim to connect transport stakeholders by enabling digital RFTM. For this reason, the consideration of DPs in association with TMSs identifies novel business service opportunities toward end-to-end digital RFTM along transport value chains.

From the extensive contributions of technological platforms and ecosystems (Asadullah et al., 2018), a market- and technology-oriented perspective on DPs is suggested to elaborate on design and governance aspects within a specific industry context (Schreieck et al., 2016). In addition, the specifications of the platform types and their technologies allow for decision-makers to explore new developments toward their digital business strategy (Asadullah et al., 2018). De Reuver et al. (2018) emphasize a need for research regarding the understanding of platform varieties in specific industries and identify three major aspects: (1) conceptual clarification of DPs and ecosystems, (2) the development of DP typology, and (3) methodical approaches to address the scope of platforms. Hence, research in the nascent field of logistics-oriented DPs is recommended for IS scholars to explore unquestioned business fields and create relevant knowledge for platform design and innovations in the context of digital ecosystems (De Reuver et al., 2018; Rix et al., 2020). Considering the above, we identify the need to develop domain-specific knowledge of DPs through the following research questions (RQs):

- RQ1: What digital platform types exist to support road freight transport management?
- RQ2: How do the digital platform types enable digital services and support road freight transport management?

Over the past years, the spectrum of existing DPs for RFTM and their digital capabilities were outlined by practitioner literature, with a focus on market organization and categorization (e.g., Baron et al., 2017; Hentschel et al., 2019; Roland Berger GmbH, 2020). Despite these efforts, grounded knowledge in a platform-driven logistics domain allows for the exploration of DPs in the context of road freight transportation. Furthermore, comprehension is gained of platform capabilities that address transport management and support service innovations based on digital technologies through a unified understanding. Although scholars have broadly examined DPs and explored platform innovations for digital business models (e.g., Dmitriev & Plastunyak, 2019; Tiwana, 2013; Trabucchi et al., 2021), more comprehensive insights into cloud-enabled road freight business are recommended to elaborate on the understanding of platforms for specific business industries (Pauli et al., 2021). To this end, a typology facilitates digital platform research activities in transport logistics by providing a domain-specific framework for the abstracted activities that RFTM tools should support. Such a typology enables researchers to gain a better understanding of the similarities and differences among RFTM functions and thus contributes to developing a common foundation for future research work. Another benefit of a typology is the provision of an overview of RFTM and its functions to practitioners, thereby supporting the selection of appropriate tools and TMS software products. Consequently, it assists transport stakeholders to define digital freight services based on DPs according to their business process requirements. Following this line of thinking, this paper develops a superordinate framework that paves the way for digital platform business in the road freight transport sector.

To answer our RQs, the remainder of this paper is organized as follows: In Chapter 2, we provide a background of DPs in transport logistics and present the platform aspects linked to the detailed activities of the RFTM process. Subsequently, in Chapter 3, we introduce our research design, which follows grounded theory research and utilizes explorative expert interviews to collect data. The findings we present in Chapter 4 constitute the identified DP characteristics and the conceptualization of a typology for DPs for RFTM. Following this, in Chapter 5, we discuss our findings and provide implications for theory and practice. Finally, we conclude our findings and offer an outlook for future research in Chapter 6.

## Domain background

### Digital platforms in freight logistics

The logistics business is greatly driven by data and provides new service opportunities due to the emergence of technological platforms. This applies particularly to the road freight sector that is yet characterized using heterogeneous and proprietary IS within a fragmented freight forwarding industry (Backhaus et al., 2017; Heinbach et al., 2021a). Therefore, the pace of digital transformation in the road freight market has resulted in different forms of platform-based forwarding business. To generate an understanding of DPs in the transport logistics sector discussed by scholars, we first aim at providing an overview of DPs with relevance to our topic.

Platform-based business in the field of transport logistics has multiple facets enabling freight capacity utilization through matchmaking demand and supply (Caplice & Sheffi, 2003; Nandiraju & Regan, 2008), facilitating information exchange by commoditized logistics services based on advanced algorithms (Witkowski et al., 2020), and supporting transport planning through the automated booking of shipments (Akac et al., 2020). In essence, DPs in the road freight transport domain represent multisided platforms (Hagiu & Wright, 2015) and can be determined as electronic marketplaces between shippers, freight forwarders, and carriers (Bierwirth et al., 2002), often referred to as electronic freight exchange (e.g., Caplice, 2007; Jain et al., 2020). Möller et al. (2019) distinguish between a digital transport marketplace and a booking platform, while a rigorous boundary of the service capabilities remains vague. Other types of multisided platforms are described in practice by freight procurement activities (i.e., resulting in tender management between shippers and carriers) (Wurst, 2020). Additionally, product platforms in the road freight transport domain exist in the form of vehicle manufacturer platforms—as investigated by Hofmann and Osterwalder (2017)—that offer telematics-based data services to road carriers for fleet management optimizations (Heinbach et al., 2022). Existing definitions, taxonomies, and systematizations from the literature that support the process of freight transportation by digital freight services are presented in Table 1.

**Table 1:** Examples of focus and applications for digital platforms in freight transportation

Source	DP Focus	Description of application in practice (Research scope)
Wurst, 2020	Tender Platform	Definition of freight tender platform in the forwarding business (Intermodal Logistics/Article for Technologies in Transport Chains)
Möller et al., 2019	Digital Transport Marketplace	Archetype for start-ups in the logistics sector (Intermodal Logistics/Digital Business Models)
Hofmann & Osterwalder, 2017	Vehicle Manufacturer Platform	Systematization for fleet management services from telematics-based platforms related to digitization in logistics (Third-Party-Logistics (3PL)/Business Model Analysis)
Bierwirth et al., 2002	Electronic Transport Marketplace	Systematization for online platforms related to freight transportation (Road Forwarding/ Framework for Electronic Marketplaces)

The view on DPs toward marketplaces in freight transportation is likewise discussed by practitioner literature, emphasizing the ability of platforms that focus on spot pricing and freight brokerage (Baron et al., 2017), matchmaking of freight shipments and load capacities (Graser et al., 2017), connectivity of IT systems that facilitate end-to-end visibility (Riedl et al., 2018), and service intermediation between shippers and carriers (Hentschel et al., 2019). Based on these contributions, we noticed a high value of granular insights from DPs for supporting increasingly digitally-enabled transport management and data-driven forwarding operations.

To illustrate the situation in practice, one may imagine a freight dispatcher that pursues to optimize utilization of freight loading resources over customer transport orders received in different data formats (e.g., paper-based, e- mail) and a fleet operator that manages the truck vehicles and transport equipment from different manufacturers using telematics technologies (Heinbach et al., 2022). These road freight stakeholders benefit from using the service offerings from DPs by receiving customer orders in a uniform format and the consolidated data from vehicles and equipment to be integrated into a “smart transport window” (Heinbach et al., 2020). But how can transport stakeholders identify the most suitable service option offered by DPs matching their needs? Given the myriad of terms applied by scholarly efforts for describing DPs in freight logistics, a service-oriented typology of technological platforms that guides the users along the process of RFTM is exceedingly helpful. This idea is reasoned by the increasing digital competition in the road freight markets — particularly in Europe — and the new economics of platform- driven markets to reduce costs (e.g., administration expenses for driver communication) along the shipment lifecycle that is subject to a digital process found in the overarching structure of transport management (Ortwein & Kuchinke, 2021). However, despite the existing research contributions that address the rise of platform markets, thereby indicating several digital service opportunities for operational users (e.g., Möller et al., 2019), more granular research of DPs capabilities aligned to the process of RFTM has not yet been presented. Thus, to the best of our knowledge, our research is the first to provide novel insights into DP markets in the road freight transport domain addressing their characteristics, data-driven services, and an understanding of technological platform varieties that support the process of RFTM.

### Role of digital forwarders

Freight forwarders ensure the movement of goods and materials from shippers to consignees via different modes of transport (e.g., air, water, land) by own transport capacities or third-party carriers. According to the legal framework (cf. §453–463 HGB in Germany), freight forwarders, therefore, contract transport orders with shippers in their own name or by involving carriers that own freight capacities to enable the required transport service, which likewise applies to logistics service providers. However, this practice occurs commonly in the highly fragmented trucking transport industry, with several hundreds of thousands of carriers in regional markets (e.g., Europe), while freight capacities are not fully utilized (Wurst, 2021, p. 31). The range of activities for performing road freight transport services generally encompasses planning, execution, and value-added services (e.g., organization, freight dispatching, real-time monitoring, billing). Furthermore, the forwarding business is traditionally a customer-specific business with tailored logistics services for customers, while price competition leads to margins of less than 2% in the truck load segments (Kille & Schwemmer, 2013, p. 23).

In light of developing platform business concepts in logistics, especially start-ups without own physical (truck) equipment began offering digital transport services to customers, focusing on data algorithms and digital interfaces centering on digital platforms to ensure digitalized RFTM operations, referred to as “digital forwarder” (Elbert & Gleser, 2019; Jain et al., 2020). For instance, the companies sennder and Instafreight operate a digital platform-based forwarding business and represent the contractual party to shippers and carriers with direct interaction, including communication. Digital forwarders map business processes digitally and act as an intermediary between shippers and carriers, providing digital freight services, such as freight pricing, transport booking, documentation, and the use of data to provide predictive services (Möller et al., 2019). The value proposition of digital forwarders is the close contact with transport stakeholders in the position of a legal freight forwarder within a fully digitalized transport workflow along freight lifecycles that offer modularized services (e.g., end-to-end shipment visibility, geo-fence notification, electronic proof of delivery) (Ortwein & Kuchinke, 2021). Hence, digital forwarders represent an emerging platform concept in logistics, enabling the digitization of transport operation activities while engaging in traditional transport management as they bypass “real” logistics service providers’ business models by transitioning established transport processes to their cloud- based environment (Dietrich & Fiege, 2017; Mikl et al., 2020). From this end, the platform-driven road freight market is complex, and diverse business concepts are developing to achieve digital road freight forwarding that supports end-to-end transport management. To understand the distinct digital platform concepts that exist in the road freight market, we believe that an approach enabling the comparison of digital platform capabilities at the transport operations level is required. This stems from the underlying concept of transport management, which presents a paradigm of activities for transport stakeholders (e.g., shippers, carriers, freight forwarders) given in practice. Hence, we explore the data-driven services of DPs and define the types based on a framework for the process of RFTM we intend to establish.

### Activities in road freight transport management

**Fig. 1:**
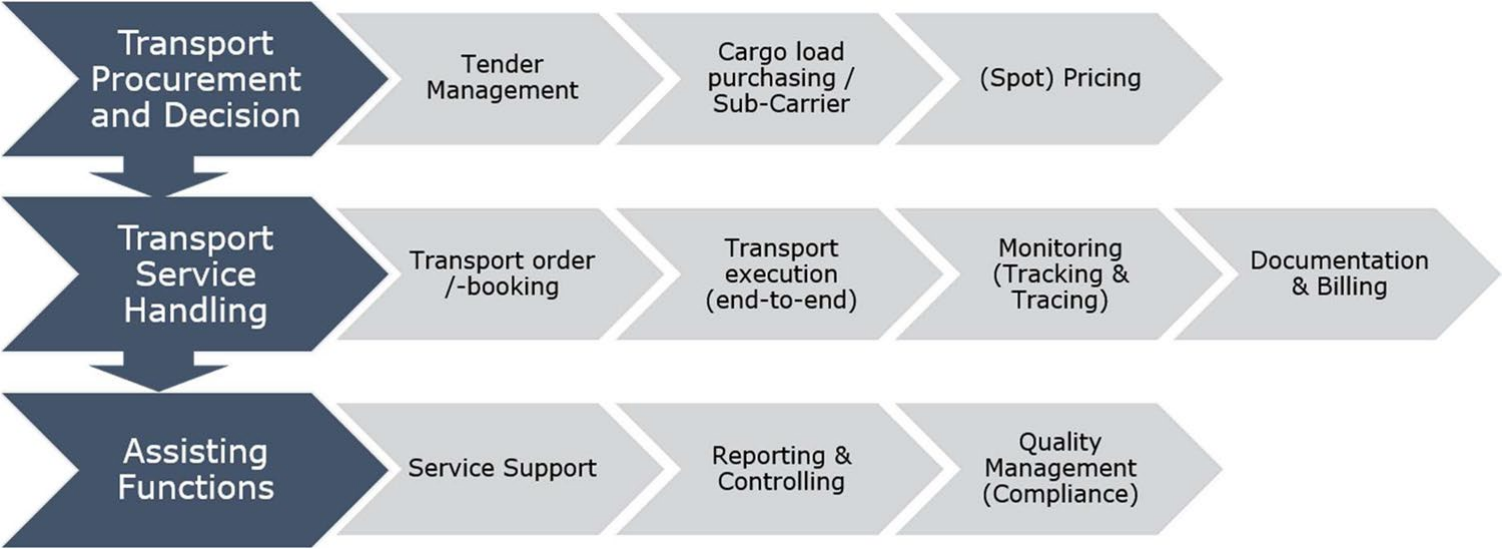
Activities of RFTM considered in this study

To explore DPs in the context of RFTM, we derive a framework from transport management activities based on Gartner’s “Magic Quadrant for Transportation Management Systems” (De Muynck et al., 2020) and Baron et al. (2017). This approach allows us to obtain a concise view of practical freight operation tasks and the relevant processes adopted by transport stakeholders to achieve an optimized workflow through IS applications in the forwarding business. For this reason, the perspective of shippers, carriers, and freight forwarders is addressed. According to De Muynck et al. (2020, p. 1), “TMSs generically refer to the category of software that deals with the planning and execution of the physical movement of goods across supply chains.” Hence, the core activities of TMS applications provide an accepted paradigm for platform research to explore the data-driven service potential for supporting transport management. Shippers are consequently enabled to plan, execute, and track shipments, while a TMS can be integrated into their enterprise resource planning (ERP) system to process orders more efficiently in a multimodal manner (Rousse, 2020). Moreover, TMSs have their origin within the scope of freight forwarder software to manage complex transport operations by assigning customer shipments to fleet resources in the trucking business. By merging the views on transport activities derived from TMSs, we develop a frame for RFTM activities that forms the basis for considering DP types and their service offerings to support transport management (e.g., matchmaking of transport orders with freight resources). Due to the high degree of specialization in the logistics sector, the emergence of DPs yields specific platform concepts that cover certain activities in the frame of RFTM. Figure 1 summarizes three transport management stages encompassing 10 activities for RFTM that we have identified for the analysis of DPs related to our topic.

First, transport management for shipments begins with the procurement of transport services by contracting service providers through tender contracts. If shippers procure transport capacities without a contract, the pricing of on-demand capacities from the market occurs on the “spot.” Second, the transport service is handled, which incorporates the planning and operationalization of freight resources. This phase involves the booking of transport services (e.g., transport order), transport execution (e.g., truck dispatching), monitoring activities (e.g., tracking and tracing of freight equipment and trucks), and documentation including billing (e.g., proof of delivery and payment). Third, assisting functions address the service support (e.g., cargo claim in case of damages), reporting and controlling (e.g., statistics for accuracy of on-time deliveries), and the management of quality and compliance aspects (e.g., management of legal certificates). The aforementioned activities are related to the freight forwarding industry (i.e., transport packages that exceed a certain weight or volume, such as the Euro-pallet considered as freight unit) in the B2B market and address heavy trucks, focused on by this paper. Based on the aligned business activities from TMSs, we further explore the digital capabilities of DPs in supporting digital RFTM and elaborate upon our research approach in the next chapter.

## Research design

In the introduction, we emphasized the emergence of DPs specifically for the road freight transport market due to their growing business potential to support transport management. These platform-enabled services correspond to the business process of TMSs products, leading to novel business models in a complex and competitive business environment. To answer our research questions established in the introduction, we aim to explore existing DPs and support transport stakeholders for the realization of digital RFTM, particularly respecting evidence from practice. Consequently, to analyze unknown platform-enabled processes (i.e., digital fleet operations to manage truck vehicles and equipment based on telematics technologies) and relevant interactions, we opted to pursue a qualitative approach by following the ideas of grounded theory research to explore the platform types and their digital capabilities (Glaser, 1978; Glaser & Strauss, 1967). The grounded theory methodology allows researchers to develop a “theory” based on rigorously collected data from qualitative resources to derive an “explanation” of a complex phenomenon. To this extent, the units of analysis are the domain-specific digital platforms according to their platform-based services, facilitating interactions between transport stakeholders to realize digital road freight transport management. Our research process follows the grounded theory concept of Strauss and Corbin (1990) since scholars benefit from greater flexibility and the use of additional knowledge to develop appropriate theories, concepts, or properties. Furthermore, grounded theory is a well-established methodology for qualitative research, particularly in logistics and supply chain management (Denk et al., 2012; Papert & Pflaum, 2017; Randall & Mello, 2012). The objective of our exploration is not to define dimensions and characteristics that are mutually exclusive yielding taxonomies (Nickerson et al., 2013).

In other words, our qualitative research design supports the conceptualization of DPs leading to typologies that classify the objects of a certain research area for gaining specific knowledge (Lambert, 2015).

## Data collection

To collect qualitative data from multiple sources, we conducted in-depth expert interviews as the main source within the framework of grounded theory research. The sample for our exploratory study is comprised of 11 associated practitioners at the management level of companies that operate digital platform businesses dedicated to the road freight transport industry. To create a sufficient database, we opted to select suitable interview partners by using the concept of theoretical sampling based on the principles of grounded theory. We selected experts from companies based on the observed varieties, addressing the platform business scopes (e.g., order-based, truck-based), freight types (e.g., dangerous goods, full and part load), range of digital processes (e.g., freight order processing, vehicle tracking), and their diversity in terms of company size, country of operation, and service specialization to create value for end-to-end digital RFTM. Associated practitioners were qualified for the interview by the authors due to their work experience, management position in the company, and the level of knowledge related to our topic observed from public information (i.e., press release). Table 2 provides an overview of our sample, including the job title of the participants, their respective work experience, and a description of the organization, the size, and the core service of the platform whereby further details cannot be presented due to confidentiality agreed with the experts respecting their business in an emerging market. During the conversation with the interviewees, we asked follow-up questions to gain more in-depth information on specific topics, for instance, business models. This strategy supports the consideration of enriching information to the purpose of our analysis, which results from discussions with the respondents (Patton, 2015), apart from the potential theoretical contribution to already-existing concepts (Morse, 2007).

**Table 2:** Overview of experts participating in the interview (anonymized for confidentiality)

No.	Organization pseudonym, size*	Digital platform description	Expert position	Work experience (years)	Interview duration
1	TRANSPARENT, small	Real-time tracking platform for trucking and courier services	Managing Director	5	0:45 h:min
2	CARGOCONNECT, small	Online platform for B2B forwarding and courier services	Managing Director	6	1:10 h:min
3	CARGOCLOUD, large	End-to-end solution provider in the freight forwarding industry	Key Account Manager	31	1:28 h:min
4	SHIPFORWARD, small	Web-based logistics software provider for SME truck transport companies	Managing Director	7	0:54 h:min
5	DIGITALTRANS, large	Global freight platform for SME transport companies and shippers	Senior Account Manager	4	0:59 h:min
6	TRUCKAHEAD, large	Workflow-software for freight forwarders and other industries	Key Account Manager	9	1:00 h:min
7	NEOLOGISTICS, medium	IT service specialist for European truck logistics	CEO	8	1:53 h:min
8	VISACARGO, medium	Multimodal visibility service platform for real-time information	Pre-Sales Manager	14	1:21 h:min
9	STEPFORWARD, small	Web-based compliance software provider for freight forwarders	Managing Director	15	1:00 h:min
10	ROADSHIPPER, large	Freight technology and IT service provider for transport systems	Sales Director	5	1:05 h:min
11	MOVINGSTAR, large	Logistics platform provider for telematics systems	Head of Sales, Marketing, Support	18	1:09 h:min

*Size according to EU definition, see: https://ec.europa.eu/growth/smes/sme-definition_de (Retrieved 18 March 2022)

Since the global road freight forwarding industry operates under different laws (e.g., hours of service regulations, load securing) and freight handling procedures follow different transport modalities (e.g., Euro-pallets are an accepted load aid for exchange among European countries), our sample of experts consisted of platform providers operating in the European road freight market to ensure a consistent data set grounded within the same legal framework for transport operations. Within the identified organizations, we attempted to acquire responsible senior experts involved in digital transport management with far-reaching experience in the freight forwarding industry who address customer processes, business developments, and technological innovations. This approach allowed us to cover a broad variety of platform-enabled services for digital RFTM, respecting the complexity of road freight processes between transport stakeholders within a fragmented business environment.

The timeframe for the interview inquiries was from July 2020 to October 2020. This lengthy period resulted from a time-consuming process of identifying targeted participants within organizations and substantial delays caused by altered availabilities during office hours due to the persisting coronavirus situation. Nevertheless, the additional time enabled us to perform iterative data collection and analysis performed by several authors of this paper. Our specific topic focus narrows the empirical basis related to platform providers. However, after conducting 11 interviews, we recognized a sharp decline in the amount of additional information that would yield new insights related to our topic. The number of interviews we performed complies with the common numerical boundaries assigned for grounded theory by scholars (Goulding, 1998; Riley, 1996). Furthermore, the collected data appeared to be sufficient for exploring digital platform types and digital services for RFTM, indicating a theoretical saturation during analysis. The interviews were conducted in the native language of the informant via web-based online conference systems and by phone since face-to-face interviews were not possible, with conversations lasting between 45 and 113 min. The interview data were then audio-recorded (Riley, 1996) and transcribed verbatim by an uninvolved typist (Ryan et al., 2009) with a background in road haulage, global freight forwarding, and logistics. With this approach, we prevented biases through data familiarity from conversations and individual notetaking by interviewers and ensured a consistent transcription following the straight conversations. Since we conducted the interviews in the native language of the informants, a translation was required to make the data available to a wider audience. Moreover, anonymity was promised to the participants to ensure confidentiality in protecting competitive-relevant information and secret knowledge. Thus, only pseudonyms for organization names are provided in this research.

To conduct the interviews, at least one author of this paper with sensitivity in the field of digital logistics and the freight forwarding industry, obtained from professional experience in that business domain and by reading literature, performed the conversations with the respondents, subject to the further development of the research process (Strauss & Corbin, 1990). An interview guide was shared with the interviewees in advance for moderation by the investigators. We followed the concept of the dramaturgical model suggested by Myers and Newman (2007) and considered their seven guidelines to organize and conduct the qualitative interviews diligently. Since the interview process must be carefully conceptualized, this model guided us to collect comprehensive data from informants related to our topic. Prior to the conversations, we provided a short introduction to the research topic of digital platforms and data-driven transport logistics by emphasizing the current trend of digital transformation, followed by the presentation of the interviewee to understand the position within the organization. Subsequently, we presented our research project and proposed 16 questions in the interview guide, encompassing the following four sections:

- Range of digital freight services offered by the platform, particular user groups, customer segments, and assignment of service to a specific logistics system (procurement, warehousing, distribution)
- Identification of technologies applied to generate services, relevant data used, service innovations in progress, and service support of end-to-end RFTM activities
- Data-driven RFTM along transport chains, including the potential of data for objects, compliance aspects to support carriers, requirements for end-to-end transportation, and impact on service quality
- Concluding remarks regarding risks and opportunities for digital freight service providers acting along the transport chain for commercial road haulage

Appendix Table 5 illustrates the questions we developed per section for the interview guideline. In addition, sketches were used to demonstrate the individual activities of RFTM in practice, presented in Section 2.3 (Fig. 1), to collect the relevant data of digital freight services offered by platforms with an assignment to the business process of road freight transportation from TMS products. Moreover, these guiding questions were introduced gradually, leading to an open and interactive discussion to discover digital platforms comprehensively supported by the domain-specific expertise of investigators. In addition, we intended to leave room for the thoughts and ideas of the respondents based on the different experiences within the scope of digital transport management in the freight forwarding industry. Hence, further discussion beyond the guided conversations was assisted by planned prompts to extend conversations, while the use of floating prompts allowed the investigator to unobtrusively draw the attention of informants toward detailed aspects and key terms (McCracken, 1988).

## Data analysis

For the coding procedure, the process of open, axial, and selective coding was applied (Strauss & Corbin, 1990). During the coding process, at least one author and one scientific assistant analyzed every sentence in the transcripts line-by-line independently to identify key concepts comprising important key characteristics and services of digital platforms (open coding). Moreover, the content related to important concepts of DPs for RFTM, particularly focusing on the digital service offerings to support transport management, was marked. Subsequently, we divided the analyzed content into smaller fragments and aggregated these into more abstract, conceptual categories using descriptive codes to label specific digital platform aspects. The focus lies on a more detailed description of the platform characteristics and digital services assigned to the individual activities of RFTM (cf. Section 2.3). To this end, the results from the coding activity were collected in memos by the authors and further aggregated to obtain characteristics of DP in the context of RFTM. We further consolidated our findings into a matrix table according to the coding dimensions by comparing the results and ensuring inter- coder reliability. While we operated independently when interpreting the results, the derived concepts and categories without coherence were reviewed and discussed until a consensus was reached. The investigators consequently connected the identified categories and concepts from open coding in terms of their relationships following a process-oriented scheme (Strauss & Corbin, 1990) with the aim of specifying categories and concepts that respect the impact of conditions (market), context (RFTM activities), related strategies (services), and consequences from strategies (axial coding). Based on the iterations between open and axial coding, the authors assigned the concepts and categories to the parts of the process-oriented scheme to address the activities of RFTM, with consideration given to all identified digital platform types. In Appendix Table 6, we present examples of the coding procedure adopted in this study.

Finally, we developed a synthesis from the identified categories, relationships, and memos (selective coding) (Strauss & Corbin, 1990). Considering the theory, the aim is to identify, group, and summarize “core” categories by investigating the relationship among one another and refining the linkages to RFTM activities. It is noteworthy that this step was challenging, as the research process required the integration of respective categories to arrive at the targeted typologies and did not involve the remaining amount of data from the transcripts (Strauss & Corbin, 1990). However, the authors performed this step intuitively, parallel to the data analysis, and focused on the exploration of platform types and related digital services.

## Findings

### Characteristics of DPs for RFTM

From the qualitative analysis, the authors derived a comprehensive range of elements characterizing the digital platforms in a specific business context. A variety of definitions, frameworks, and representations of business models exist, namely the VISOR framework (El Sawy & Pereira, 2013), the Business Model Canvas (Osterwalder & Pigneur, 2010), and the Business Model Navigator (Gassmann et al., 2014). Most elements used in these frameworks are similar. Due to the explicit coverage of the element “logistics platform,” we have selected the VISOR framework (El Sawy & Pereira, 2013). Our results for the final characteristics of DPs are presented in Table 3 and encompass 14 dimensions assigned to the five components of the VISOR framework (El Sawy & Pereira, 2013): value proposition, interface, logistics platform, organizing model and revenue model.

**Table 3:** Dimensions and characteristics of DPs for RFTM

	Dimension	Characteristics					
Value Proposition	(1) Visibility Service	Vehicle and Driver	Shipment	Load	Freight Rate	Document	
	(2) Optimization Service	Communi- cation	Order to Payment	Asset Utilization	Quality and Compliance	Delivery Time	Route
	(3) Analytical Service	Delivery Performance	Transport Cost	Capacity	Resources	Environment	None
Logistics Platform	(4) Data Resource	Order Data	Geo Data	Vehicle Data	Traffic Data	Driver Data	External Data
	(5) Data Source	User	Mobile Devices	Tracking Devices	Telematics	External Systems	
	(6) Data Activity	Data Generation	Data Processing	Data Exchange	Data Transmission	Data Aggregation	
Interface	(7) Interface Type	Mobile Application			Web Application		
	(8) Data Interface	API	JSON	CSV	XML	Flat File	
Organizing Model	(9) Platform Boundary	Shipper-to-Carrier			Carrier-to-Carrier		
	(10) Order Type	Spot		Recurring		Contract	
	(11) Load Type	Full Truck Load (FTL)	Part Truck Load (PTL)	Less than Truck Load (LTL)		Groupage	
	(12) Connectivity	Shipper	Consignee	Logistics Service Provider and Forwarder	Carrier	Subcontractor	
Revenue	(13) Profit Basis	Pay-Per-Use	Subscription	Freemium		Offer-based	
	(14) Price Basis	Per Vehicle	Per User	Per Load	Per Shipment	Per Tour	Fixed Rate

We follow the choice of non-exclusive characteristics as advocated by Möller et al. (2020) and visualize our finding as a morphological box (Ritchey, 1998). This proceeding is appropriate since an exclusivity of results may lead to complexity and impede a clear typology. Within the morphological box, we present all the characteristics we have gathered to constitute the elements of DPs. Some characteristics were not assigned to any dimension and remained unknown, and we decided not to include them to obtain clear results for further typology. The word “none” is assigned if a platform can operate without specific dimensions. The decomposed view on the DPs allows us to present a holistic view and achieve an overarching understanding of the DPs for RFTM purposefully. For the DPs examined in this paper, the relevant characteristics are reflected by a set of different combinations with different characteristics.

### Value proposition

It was found that participants of DPs have different reasons for using a service. Our research identified three service dimensions, derived from the interviews. We exemplify the findings with the respective statements elicited from single interviews:

“The core of our platforms is to provide an overview of truck capacities and freight loads available in the transport market.” (CARGOCLOUD)

The (1) visibility service offered by DPs comprises various characteristics. In DPs, visibility is enabled by tracking systems (i.e., telematics and mobile apps) and shared information on the web for vehicle and driver, shipment, load, freight rate, and document. The generated transparency refers to real-time notifications (e.g., estimated time of arrival, ETA) during the transport progress. Documents, such as proofs of delivery (POD), waybills, and other shipment-related papers, are transmitted digitally and can impact subsequent activities (e.g., billing process) in transport chains (i.e., an uploaded POD by the driver via a mobile app can automatically trigger the payment process). Another value dimension is related to (2) optimization service, for which we observed six characteristics:

“We support our customers by accelerating the invoice creation process (...). Moreover, we offer an accounting service.” (TRANSPARENT)

This element encompasses communication, order to payment, asset utilization, quality and compliance, delivery time, and route. Optimization is created by enhanced individual communication between the actors (e.g., truck dispatcher and driver) via mobile apps. Trucks and transport equipment are managed by carriers as physical assets, and load capacities are efficiently utilized by load-matching services. Quality comprises on-time delivery performance, while compliance refers to legal statements (e.g., cargo insurance). Real-time incidents (e.g., accidents during truck operation) impact the delivery time, and these events are derived from integrated route- planning services. Furthermore, DPs support truck navigation through the dedicated routing of special cargo (e.g., dangerous goods). Next, (3) analytical services comprise various technologies, such as machine learning, intelligent algorithms, and artificial intelligence (AI) with several areas of application:

“To measure the carbon footprint of freight packages, we collect a high number of data points.” (CARGOCONNECT)

The dimensions addressed by the analyzed DPs comprise delivery performance, transport cost, capacity, resources, and environment. Some platforms grant shippers the option to set target values for transport lead times on certain transport lanes and receive ranked results about carriers, similar to a benchmark analysis.

Whereas capacities (e.g., cargo space or trucks) and resources (e.g., driver time) are addressed by DPs, a nascent aspect for analytics is the environment, targeting the emissions resulting from truck transportation based on the fuel consumed or the carbon emissions generated by the trucks. This aspect is particularly important for the recent taxation on CO2 for transport activities in the European road freight transport domain since DPs are recognized as an enabler to support emission performance from freight operations shared with customers.

### Logistics platform

Data is the key element of platform models and enables the services provided. For data, we have identified three categories:

“We (…) see ourselves as an enabler of services (…) while the basis for our services is comprehensive geodata.” (VISACARGO)

The first category is defined as (4) data resource and describes a set of value-creation types, including order data (i.e., weight and volume of shipments), geo data related to the positions of entities such as trucks via GPS, vehicle data (e.g., fuel consumption or tire pressure), traffic data and driver data, such as driver times from digital tachographs built-in trucks. Last, external data refers to resources from other systems maintained in the cloud, such as web-based loading equipment administration (e.g., Euro-pallets). The (5) data source identified in our research entails five characteristics: user, mobile devices, tracking devices, telematics, and external systems. Users represent the platform participants and provide shipment data. Mobile devices deliver transport details from drivers during transport operation, such as completed transport orders upon truck arrival or transport instructions received. Meanwhile, tracking devices are related to electronic positioning devices (i.e., mobile trackers facilitating GPS). Furthermore, telematics is a technical system that interacts with electronic control units inside trucks and allows diagnostical data to be transmitted. Finally, external systems refer to IT systems and the data compilation with relevance to transport operations from shippers or freight forwarders. In our study, we have identified five different types of (6) data activity: First are data generation and data processing (i.e., positions from driver mobile applications). Meanwhile, data exchange refers to automated data communication between systems. Moreover, the data transmission addresses the bi-direction of data from systems in a DP (e.g., between ERP and TMS). Finally, data aggregation occurs if data is gathered from different sources and integrated into a single platform. A holistic focus on IT interfaces is required if data are exchanged, transmitted, or aggregated and involves different IT systems.

### Interface

Interactions between the platforms and participants necessitate interfaces for which we detected two categories:

“Internally, we call the service module that we offer API generalization. This means that we try to integrate all telematics systems that are available on the market and offer their services via our platform.” (MOVINGSTAR)

The (7) interface type can be distinguished as mobile application and web application. The application of driver apps is of considerable help, particularly for freight dispatchers and fleet managers to get and remain in direct communication and provide transport progress related to real-time information and changes for operations that may occur (e.g., order details, truck issues at places of un−/loading). A (8) data interface is essential to enable the data handling between different IT systems (e.g., TMS, telematics, loading aid system, accounting system). The types of data transmission refer to Application Programming Interfaces (API), JavaScript Object Notation (JSON), Comma-separated Value (CSV), Extensible Markup Language (XML), and Flat File. However, the authors noted that the examined DPs either provide extensive APIs that encapsulate the data or use simple formats such as JSON as a technical interface for transport orders:

“We receive the majority of orders via an SAP API, but many customers also send us the data in a JSON file.” (CARGOCLOUD)

### Organizing model

The organizing models provide details on the structure and process of the platforms required to realize the services:

“Our platform users are the road carriers since we’d like to know when and how much truck load capacities will be available connected to the shipper side that provide the details of a full or part load to be carried.” (NEOLOGISTICS)

The (9) platform boundary is linked to the target participants and their organizational business structure: shipper-to-carrier (vertical) and carrier-to-carrier (horizontal). (10) Order type determines contractual aspects and addresses transport orders related to spot, recurring, or contract. Transport orders are created on spot without a binding obligation from transport service providers and are often related to infrequent freight shipments with low shipping volumes. Typically, contracts are connected to tender platforms, and shippers invite carriers to receive freight bids and freight rate quotations for higher cargo volumes mostly based on fixed transport lanes. Meanwhile, recurring is an option that relates to frequent freight loads. Identified characteristics for (11) load types are full truck load (FTL), part truck load (PTL), less thank truck load (LTL), and groupage. These types describe the shipment volume and the truck loading format focused on by the platform participants.

In particular, FTL services are managed directly “door-to-door,” whereas groupage shipments require additional handling in warehouses to consolidate them into larger loading volumes, such as PTL. The (12) connectivity spans the group of capable service recipients, namely shipper, consignee, logistics service provider and forwarder, carrier, and subcontractor. The carrier remains the key transport service provider of DPs due to the carrier’s ownership of physical (truck) assets and the direct integration of “real” freight capacities.

### Revenue model

A financial income for the DPs from the conducted in-depth interviews reveals two revenue models:

“In addition to fee-based information and services, we also offer our customers a service free of charge, which, however, only includes basic information, for example, about deliveries.” (MOVINGSTAR)

For the DP, a (13) profit basis is associated with pay-per-use, subscription, freemium, and offer-based options, and these concepts indicate the platform strategy for profitability. The units for generating the revenue are reflected by the (14) price basis. Herein, services from DPs are charged per vehicle, per user, per load, per shipment, per tour, or as a fixed rate.

## Typology of DPs for RFTM

**Fig. 2:**
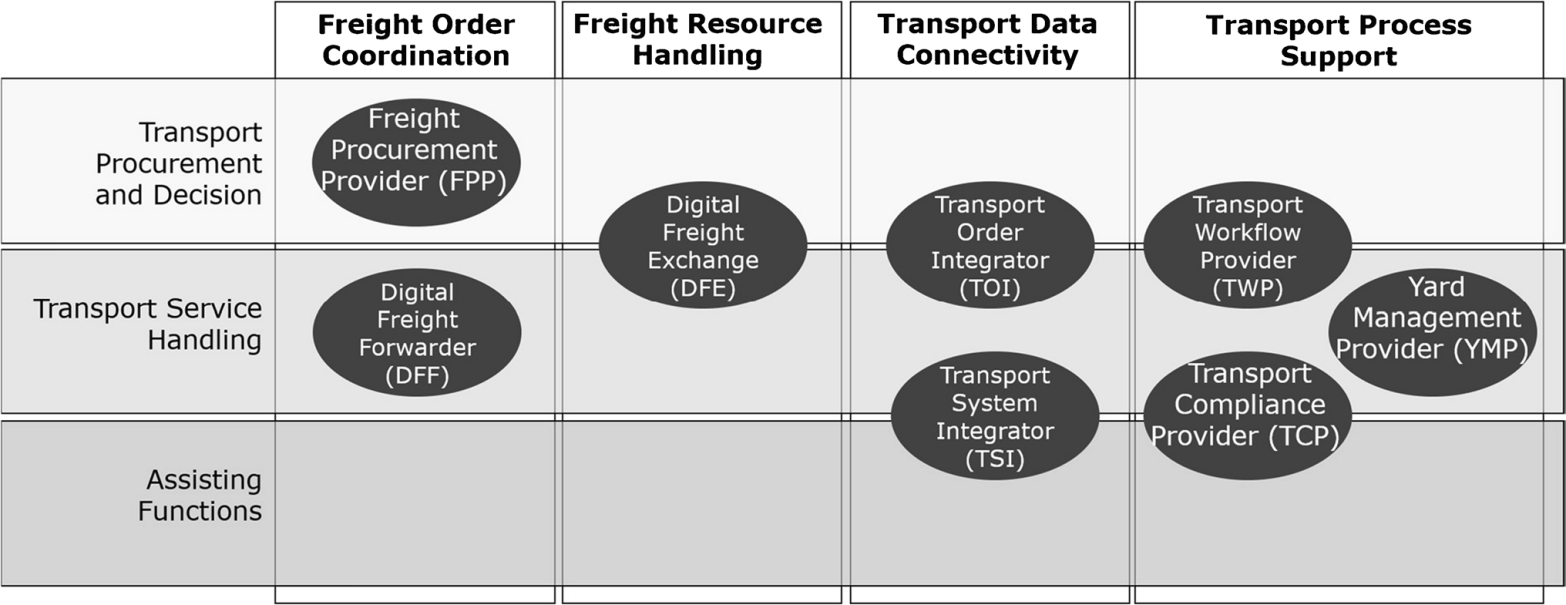
Proposed set of DP types for RFTM

From the analysis of the data, we conceptualized eight types of DPs and further clustered them into four main categories according to the scope of platform-enabled service offerings to support digital RFTM we have explored. These main categories are freight order coordination, freight resource handling, transport data connectivity, and transport process support (Fig. 2). We further assigned the three transport management stages introduced in Section 2.3, including transport procurement and decision (e.g., purchasing of load capacities), transport service handling (e.g., transport order handling), and assisting functions (e.g., quality assurance).

An overlap of DPs between the stages was noted due to the range of digital freight services provided by a DP, demonstrating relevance for several stages (i.e., the combination of freight procurement and order management platforms). Additionally, we observed that one platform provider can operate various platforms, leading to different platform types for digitizing RFTM activities. This was identified particularly from the interview with CARGOCLOUD, which operates a multi-platform strategy facilitating a more comprehensive digital service approach to cover the different main categories throughout the transport management process (i.e., from freight procurement to data exchange between TMS applications).

In Table 4, we present an overview of all DP types identified in our explorative study. To illustrate the identified participants that take part in a DP, we apply the notation “x2y” introduced by Bierwirth et al. (2002) to constitute the relationship by defining “x” as the service supplier and “y” as the demanding party requesting the same services vice versa via the DP. The nomenclature is to be read as follows: “S” for the shipper, “C” for the carrier, and “F” for the freight forwarder. The interviewed organizations are presented in a separate column by number according to the overview of participating experts presented in Table 2. Since a single organization from our interviews might operate more than one platform solution, one organization can be assigned to multiple DP types.

**Table 4:** Overview of main categories and typology derived from DPs for RFTM

Main category	No.	DP Typology	DP Description	DP Participants
Freight Order Coordination	3,10	Freight Procurement Provider (FPP)	• Sourcing of transport capacities for shippers (e.g., tender contracts) • Online freight auctioning for invited participants • Transport order management for fixed transport lanes	C2S, F2S
	2, 7	Digital Freight Forwarder (DFF)	• Processing of transport orders including freight brokerage • Virtual logistics service provider offering a range of digital services • Instant quotations for spot orders through ad hoc pricing	C2S, C2F, F2S
Freight Resource Handling	3, 5, 10	Digital Freight Exchange (DFE)	• Intermediary between demand and supply for freight orders • Matching of freight capacities and loads (e.g., best price) • Profiling of participants allows for individual services	C2S, C2C, F2S, F2C, F2F
Transport Data Connectivity	3	Transport Order Integrator (TOI)	• Integration of external transport order systems • Data exchange between different TMS applications • Seamless tracking and tracing throughout transport chains	C2S, C2F, F2S
	8, 11	Transport System Integrator (TSI)	• Operational transport data integration from TMS and telematics systems • Single point of data access concept to advanced asset monitoring • Streamlined data aggregation to delivered analytical services	C2S, F2S
Transport Process Support	1, 4, 6	Transport Workflow Provider (TWP)	• Transport visibility and event notifications for transport participants • Individual and modular workflow assignment toward driver • Transport order assistance for registration and communication	C2C, F2C, C2S, F2S
	10	Yard Management Provider (YMP)	• Estimated time of arrival (ETA) notification to manage gate activities • Synchronized information flow between asset, gate, and resources • Automated resource allocation based on immediate geo-positioning	C2S, F2S
	9	Transport Compliance Provider (TCP)	• Legal compliance and monitoring of transport activities • Surveillance of individual requirement for subcontracted carriers • Legal status is indicated by certificates and generates notifications	C2S, C2F, F2S, C2C

The category freight order coordination identified by the authors encompasses platform providers that contribute to the handling and coordination of freight orders:

“We have both large and small customers, which we have acquired, in part, through tenders (...). As a result, we receive loads every day and redistribute them through our carrier network.” (NEOLOGISTICS)

Freight purchasing services are presented by freight procurement provider (FPP) and the tendering of freight orders through fixed contracts with carriers and freight forwarders for individual transport lanes (CARGOCLOUD, ROADSHIPPER). Digital freight forwarders (DFFs) are detected by service-oriented DPs in the transport chain, and providers offer digital services related to the management of freight orders mainly to shippers as the owners of cargo and decision-makers for transport orders (CARGOCONNECT, NEOLOGISTICS). Freight resource handling describes a DP with the ability to exchange and transmit freight information following the cloud paradigm of a marketplace:

“At the heart of our business, we offer our customers a digital open freight marketplace. On this marketplace, shippers can publish transport requests, and transport service providers can submit offers.” (DIGITALTRANS)

The authors found that a digital freight exchange (DFE) reveals matching capabilities for freight loads and cargo capacities according to a “best match” or “best carrier” concept (CARGOCLOUD, DIGITALTRANS, ROADSHIPPER). The DP aims to utilize loads and capacities to attain attractiveness for shippers and transport service providers related to costs. The integration of transport-related data from different data sources leads to a DP category conceptualized as transport data connectivity:

“As I said, the platform is operating in the center, the vehicles are connected to it and [company name anonymized] and [company name anonymized] are now offering their own services via our platform.” (MOVINGSTAR)

A transport order integrator (TOI) focuses on the order information and aggregation of data via interfaces from external systems with a focus on order monitoring and shipment tracking and tracing (CARGOCLOUD). Similarly, a transport system integrator (TSI) forms a dedicated DP type that aggregates operational transport data from several IS and digital information technologies in road trucks (VISACARGO, MOVINGSTAR). A specific variety of DPs was identified by the authors leading to the category transport process support, which forms the largest group of platform types for RFTM:

“Our truck router planner enables efficient route planning.” (SHIPFORWARD)

A transport workflow provider (TWP) offers end-to-end visibility services and tools to improve order workflow and related RFTM activities (TRANSPARENT, SHIPFORWARD, TRUCKAHEAD). For example, TRUCKAHEAD has developed a mobile app to enhance communication (e.g., between the freight dispatcher and driver), manage fleets efficiently (e.g., truck maintenance issue between the driver and fleet manager), and automate processes (e.g., event-based notifications, checklists for instruction). To improve the coordination of transport activities on customer premises, a yard management provider (YMP) addresses the efficiency of gate-in and gate-out processes and the reduction of idling time via the synchronization of transport order information between a transport service provider and consignees based on ETAs (ROADSHIPPER). Other than the operational efficiency, we identified a transport compliance provider (TCP) as a special platform type that enables compliance monitoring of carriers and subcontracted partners along transport chains (STEPFORWARD). In addition, carriers can establish processes related to audits and self-evaluations to ensure full transport compliance with shipper requirements that facilitates the connection of compliance profiles with shippers’ transport decisions (i.e., during freight purchasing or the tender process).

## Service assignment of DP types for RFTM

In our analysis, we observed that a specific organization can operate multiple DP types. The contributions of a certain DP type are provided by more than one organization. The eight platform types we conceptualized were not present in all 11 interviews we analyzed. We identify the differences in DP types by assigning the various data-based service capabilities aligned to the RFTM activities presented in Section 2.3 (Fig. 1). To distinguish between basic and advanced digital services, we abstracted the service configurations based on the service offerings identified from DPs:

“We ensure that the data we generate or receive, for example, arrival times are prepared in such a way that they can be used by other planning systems.” (VISACARGO)

**Fig. 3:**
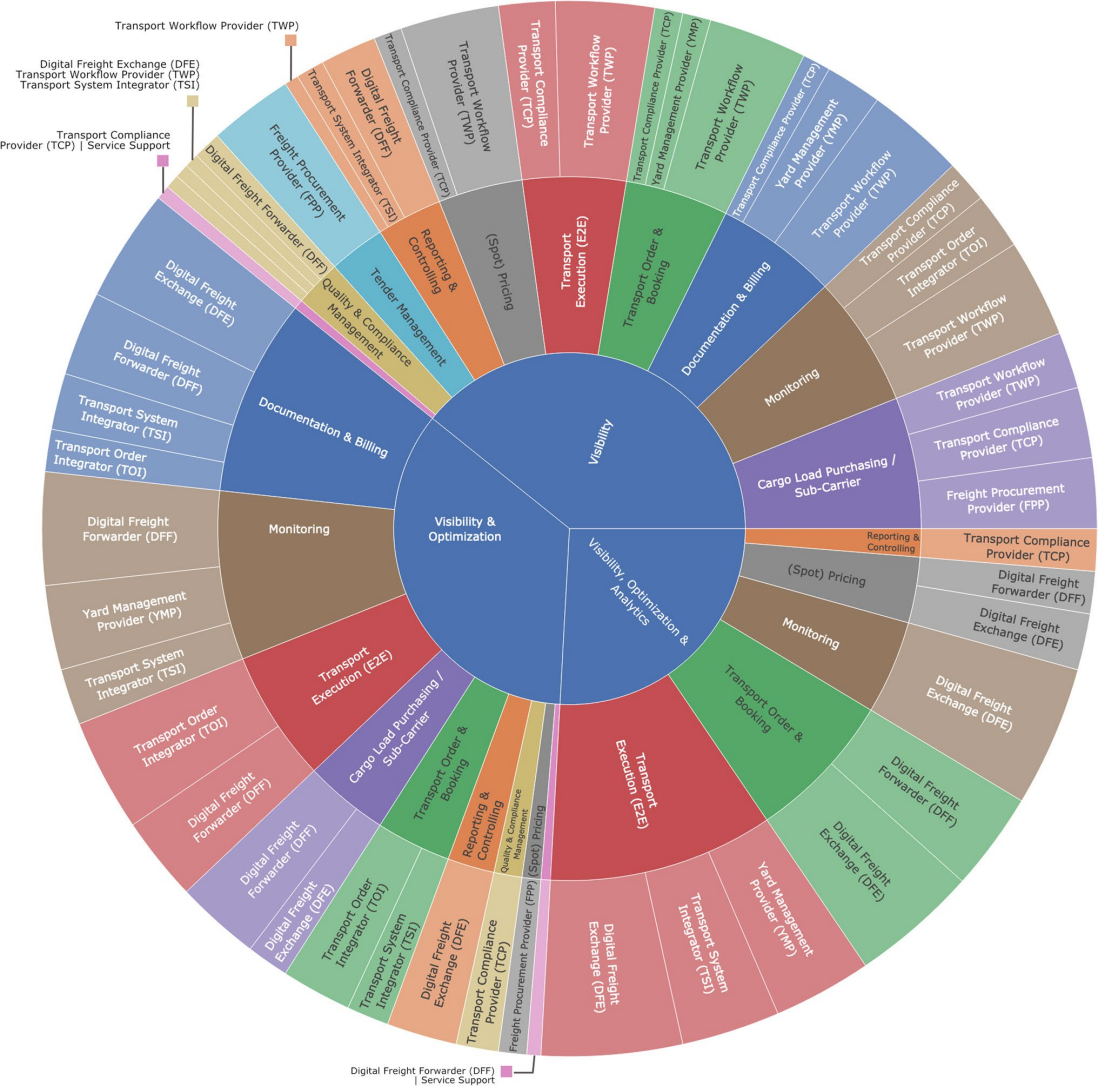
Assignment of services to DP types for RFTM activities

For this reason, the coded content from interviews was aggregated, and we assigned the digital services to the dimensions of value proposition comprising visibility (V), optimization (O), and analytics (A). To illustrate the individual service configurations of DPs based on the expert interviews, we further assigned the data service capabilities along the individual stages of the RFTM process (cf. Section 2.3) by compiling a sunburst chart to master the multidimensional perspectives (Fig. 3). Through data visualization, we captured the digital service sets (e.g., visibility and optimization) of DP types and present their connection to the RFTM stages comprehensively and comparably. The core is developed by the value proposition dimensions and connected to the RFTM activities presented in the middle layer of the chart. Meanwhile, the outer layer represents the final typology of DPs based on our findings (cf. Section 4.2). As a result, the figure illustrates the levels of digital maturity of the logistics platforms we have analyzed. Accordingly, the road freight transport domain is undergoing a digital transformation. Companies that have been in the market for longer periods (e.g., DFE providers such as Timocom) are already equipped with extensive services for analysis and optimization. In contrast, new participants are currently establishing the required data basis through visibility services (e.g., TWPs such as the company Habbl). In all service sets, visibility forms a key service for all DPs. Following the activities of RFTM, we noted that some are linked to one service from DP types (e.g., tender management was assigned solely to the visibility for FPPs), while other activities are connected to multiple services (e.g., monitoring and documentation and billing are assigned to visibility and optimization for DFFs and DFEs). From the illustration, we learn that the RFTM tasks of transport order and booking, transport execution, and monitoring are covered by all service sets, emphasizing their significance in the market. In addition, documentation and billing, spot pricing, cargo load purchasing, and quality and compliance management represent emerging tasks that are increasingly covered by DPs.

To this end, we found that digital services contribute to RFTM by different platforms. It is remarkable that services comprising visibility, optimization, and analytics are predominantly offered by platform providers that utilize freight data connected to the orders and resources of integrated components (e.g., physical assets, such as trucks). The corresponding services identified by the authors address the activities of transport order/booking, transport execution (end-to-end), and monitoring (tracking and tracing) (TRANSPARENT, CARGOCLOUD, DIGITALTRANS, NEOLOGISTICS, ROADSHIPPER, MOVINGSTAR). Herein, the yard management provider (YMP) provides an analytical service regarding the ETA service, particularly for the arrival notification of truck vehicles at the point of loading and unloading to support advanced yard operations and efficient gate management.

From our findings, we formulate an initial conclusion and infer that DPs can offer a greater value of digital services for RFTM to customers supporting operational transport management tasks if transport data are available from interconnected IS allowing for a streamlined flow of data across activities. That is, if data are available from order entries in the platform, an automated billing service for carriers may be additionally provided. From the organization MOVINGSTAR playing the role of a transport system integrator (TSI), we learned that carriers use DPs to receive harmonized services based on telematics systems to manage their fleet operations engaged with TMS (e.g., fuel consumption, maintenance service, tire status). This occurs without data barriers that exist for mixed fleets due to the different telematics services offered by truck manufacturers. We found that a digital freight forwarder (DFF), digital freight exchange (DFE), transport system integrator (TCI), and yard management provider (YMP) obtain operational data from shippers and carriers, and advanced analytics services are realized via data-enabled intelligent technologies (e.g., AI-based routing, an intelligent algorithm for matching, ETA prediction). Therefore, access to operational road freight data appears to be an enabler for DPs to provide advanced data-driven services for RFTM, while market maturity is key for successful service innovations.

Based on our interviews, we found tender management to be a special discipline for freight procurement providers (FPPs) since the platform generates visibility primarily for shippers and gathers freight rates for individual transport offers from carriers (CARGOCONNECT, ROADSHIPPER). Cargo load purchasing/sub- carrier DPs demonstrate visibility and optimization services and allow for the integration of third-party carriers to accelerate the onboarding process if carriers are unable or unwilling to serve a regular transport lane as requested by customers (e.g., shippers). The organization TRUCKAHEAD facilitates end-to-end communication between truck dispatchers and new drivers from other carriers using mobile apps and the platform solution. Specifically, for the spot market, transport pricing remains an attractive field for digital freight forwarders (DFFs) and digital freight exchanges (DFEs) since DPs have integrated service mechanisms to match freight requests and available load capacities that allow shippers, carriers, and forwarders to search for and offer freight rates in a non-contracted business environment. Insofar, freight rates are based on the market conditions and are applied for LTL, groupage, and recurring shipments. Furthermore, service support refers to the service quality of transport data for shippers and carriers through the enrichment of values by advanced technologies (e.g., algorithms).

While the trucking business environment in the freight forwarding industry is characterized by vigorous subcontracting relationships and underlies various regulations related to driver safety and vehicle compliance, the authors were surprised to find that only the organization STEPFORWARD offers relevant digital freight service solutions addressing pure compliance aspects. From the interview, we found that compliance plays an increasingly significant role throughout RFTM activities. Carrier compliance is ensured through profiles provided by the transport compliance provider (TCP), which include the legal aspects of conducting compliant transportation services. These legal aspects are made visible in the transport procurement and are often combined by shippers to carefully check carrier compliance (e.g., compliance of an external carrier before the transport order is ultimately assigned). However, compliance remains a delicate area in the realm of commercial trucking due to the transport liability aspects ensured by shippers throughout the subcontracted business with multiple transport operators in use. Thus, the identified phenomenon of TCP is still required to become mature.

## Discussion and implications

While the focus of this study was on operational road freight transport tasks to generate in-depth knowledge in a domain-specific research area, the aim was to investigate DPs in the real business environment. Based on the findings, we answer the two research questions and provide a basis for more in-depth investigations into the effects of DPs on the logistics market. First, we identify data service offerings from DPs currently available in the road freight market and thus create a comparative overview of DP types (RQ1). Second, we elicit the structure from the existing body of knowledge and the consolidated findings of our empirical investigation to understand the service capabilities of DPs to support RFTM (RQ2). Since our study is just beginning to explore a new market, we discuss the results against the obtained data, identify implications that can be drawn, and conclude by presenting limitations and work that is yet to be done. While this study provides insights into a hitherto under-researched emerging market, we are convinced that it can likewise contribute to the broader picture of understanding digital transformation in logistics within a specific business context. Therefore, we first highlight the linkages with a more general body of knowledge and embed our results.

Without exception, the 11 experts we interviewed identified an interaction between multiple sides as a core characteristic of DPs for RFTM. This results from underlying DPs that have established digital business models in which service providers utilize data as the primary resource to offer services to customers (El Sawy & Pereira, 2013). Our findings substantiated the perspective of DPs as multisided platforms (Hagiu & Wright, 2015) that coordinate the generation, processing, exchange, transmission, and aggregation of transport data as the input and services that provide visibility, optimization, and analytical value as the output. Subsequently, our study abstracts from these rather technical cloud service capabilities by the service dimensions of visibility, optimization, and analytics and their assignments to RFTM (cf. Section 4.3), thus allowing us to explore and compare solutions among platform providers and TMS product vendors. In line with similar views of other scholars (e.g., Gruchmann et al., 2020; Kern, 2021), we infer that a key component to the provision of DPs is the vertical and horizontal integration of information in supply chains, constituting their reputation as flexible and cost-competitive resources in road freight operations fostering advanced network effects. While the supporting concept is discussed in theory as cloud logistics (Delfmann & Jaekel, 2012), standardized modules emerge increasingly to connect both services and virtualized resources, leading to new concepts such as “cloud logistics service blueprints,” as proposed by Glöckner et al. (2020). Moreover, we expect the share of platforms offering visibility, optimization, and analytics to increase in the future (cf. Figure 3) as advanced IS gain more widespread use. Whether the current technical lead will provide a market barrier or whether platform integrations between process stages can even be expected as a result of the development would serve as an important question in the future design of digital platforms in logistics.

To this end, practical implications from our findings arise for road freight professionals, since a mutual understanding between operational teams of shippers and carriers and other transport stakeholders is established in the sphere of platform-based transport logistics. This helps to identify digital business requirements from customers and further guides decision-makers for RFTM toward the identification and selection of suitable DP providers in the market. In addition, other transport stakeholders with a natural interest in the ongoing momentum of digital transformation benefit from our findings by the framed sets of platform-based service capabilities that offer opportunities (i.e., by enriching digital service value to transport monitoring to ensure visibility of freight equipment for safe transport operations—for example, insurance companies). Foremost, the study enables decision-makers involved in operational transport management activities to gain a holistic understanding of DP service capabilities. To achieve this, practitioners are navigated through the sphere of emerging DP concepts, their types, and service boundaries in a dynamic and complex cloud-enabled road freight transport domain that increasingly intersects with the business nature of TMS products. Our typology can be used by enterprises to learn about DP business models, thus providing a basis for strategic consideration (Tönnissen et al., 2020). Additionally, transport management professionals are enabled to closely align their business structures to make make-or-join decisions regarding digital platform ecosystems (Hein et al., 2020). In effect, this could provide a common strategic ground for new joint business opportunities between shippers, carriers, and forwarders to strengthen road freight collaboration toward integrated supply by intelligent digital platforms (Yang et al., 2017) based on the business process of TMSs. Particularly for start-ups and entrepreneurs, our findings offer the opportunity for these entities to classify themselves and their competitors and learn about unexpected business opportunities and “freight technologies providers” that leverage the digital transformation of logistics based on collaborative logistics platform strategies (Heinbach et al., 2021b). Furthermore, emerging platform-based concepts for RFTM may promote carrier competition, as the platform supports transport service quality and allows both shippers and carriers to address the legal risks within connected supply chains.

For researchers, the implications of our study are derived by the conceptualized typology of DPs for transport management (cf. Section 4.2), suggesting components for an emerging platform domain (Rix et al., 2020) and following the concept of virtualized resources encapsulated in services to support logistics flows and transformation (Glöckner et al., 2020). This ties in with the existing research on the general transformation of digital businesses in the logistics market (e.g., Möller et al., 2019). Additionally, the eight platform types extend existing descriptions of platform providers based on empirical investigations performed by Elbert and Gleser (2019) with a more concise perspective on road transport activities, including the stages of procurement, handling, and assisting functions of freight transport orders. However, our analysis stylized additional facets, such as the four overarching categories and the business purpose of platforms (cf. Table 3), thus adding qualitative knowledge beyond the existing specification of platform-based business concepts toward digitalized freight forwarding primarily addressed by practical contributions (e.g., Baron et al., 2017; Riedl et al., 2018) and providing a basic understanding of domain-specific data-driven service opportunities from DPs. The results consequently help to illuminate the market and emerging platform types, such as transport compliance providers (TCP), which are capable to support legal conformity in the transport business for shippers, carriers, forwarders, and other external actors (e.g., subcontracted carriers). In essence, researchers that examine DPs benefit from our novel approach to study other domain-specific platform phenomena by defining the digital service capabilities of platforms aligned to business processes in organizations. Hence, it serves as a blueprint for scholars studying modular service architectures of DPs (Tiwana et al., 2010) while uncovering platform-based value propositions that further stimulate discussion on capturing value in digital platform ecosystems (Hein et al., 2020).

It remains to be said that our study is likewise subject to the inherent limitations of exploratory surveys. While the results offer several insights into and opportunities for new research, expert interviews cannot guarantee a complete representation of the road freight market. Further interviews, especially focusing on other regional markets (e.g., North America), could thus increase the precision of the developed structure and might modify service assignments but, in our view, would likely neither change the key proposition of the study nor achieve completeness. We, therefore, apply the two methodologically accepted measures of theoretical saturation and the number of interviews required to ensure sufficient quality. Nevertheless, the findings of our study are restricted to the European market and provide room for further explorations of niche players existing in other regional markets that meet the legal requirements and support digital transport management. Naturally, the results must be used cautiously in this respect, yet we wish to emphasize the initial implications that can be considered worthwhile from our perspective.

## Conclusions and outlook

In this paper, we investigate DPs in the European freight forwarding industry by conducting qualitative research with a focus on road freight transport management (RFTM) activities. Our exploration is grounded on in-depth expert interviews involving 11 organizations from practice in the domain-specific digital business industry. Overall, this study contributes to logistics and digital platform research by exploring and analyzing the data- driven service capabilities of DPs to support RFTM. Moreover, this study broadens the understanding of digital platforms for RFTM and sheds light on the different characteristics. To this end, we identified various characteristics assigned to 14 dimensions and conceptualized eight DP types regarding RFTM. Through the service assignments for RFTM, we demonstrated the digital capabilities of DPs for data-driven transport management (Heinbach et al., 2021a). With the assignment of services from DP types to RFTM activities, this study further contributes to practice by revealing the main areas of digital service providers and identifying blind spots, which can be addressed by companies or entrepreneurs. Moreover, our assigned services demonstrate a novel paradigm to support navigation for digital service opportunities in the realm of emerging platform markets. Hence, this study serves as a starting point for future research initiatives. Further research should investigate critical success factors of DPs for RFTM in the future and analyze details on digital freight services to enable connected transport management from the user perspective (e.g., carriers) to understand both barriers and opportunities for business applications. This could include an exploration of other platform types and further analysis to elaborate a more profound platform understanding in contrast to industrial platform mechanisms (Pauli et al., 2021). Therefore, additional data is required to provide more evidence on the services assigned to our DP types, and we recommend addressing this field with an empirical study on a larger scale. Digital business models in the freight forwarding industry should be investigated in the context of “smart service” concepts to discover the role that DP providers play to achieve “smart transport management systems,” as advocated by Stefansson and Lumsden (2008). Technologies related to vehicle and infrastructure require scholarly efforts to reveal innovative platform concepts toward a smart forwarding industry as envisioned by (Heinbach et al., 2020) that could address the combination of different DPs and act as a meta-cloud paradigm for digital transport operations.

In essence, additional investigations, including the design and development of a prototype, are required to unveil the capabilities of data-driven platforms within digital freight ecosystems. We are confident that the insights obtained by our study will assist future academic researchers who wish to investigate practical platform applications or develop platform ecosystem frameworks for testing platform-based service theories in the context of freight transportation and logistics. While the goal of this paper was to create awareness and explore an emerging research object in a vital business area, we hope to gain the interest of other scholars regarding digital platform innovations in transport management, aiming for sustainable and shared data-driven service developments in an inter-connected road freight transport domain.

## Appendix 1

**Table 5:** Sections and questions of the interview guideline

A. Categorization and general questions	
1.	What digital services (DS) does your company offer for the players in the transport chain within commercial road transportation?
2.	Are there any specific user groups and sectors you are focusing with your DS in the context of commercial road transportation?
3.	Which of the logistics systems are addressed by the DS you are offering to customers?
B. Technology, data and digital services	
4.	What are the analytical methods and technologies in place in order to offer your customers intelligent solutions?
5.	Which type of data are relevant for the delivery of DS to customers?
6.	Are there any additional platform-based services, you are offering to customers already, or you are planning to offer them in future?
7.	What is your understanding of a fully connected “end-to-end” (E2E) transport chain?
8.	With reference to the E2E transport chain: which activities for transport management are being supported by your DS?
C. Data-driven added value along the transport chain	
9.	How do you assess the potential of data-driven value-added process in real time by an object-oriented and autonomously managed transport?
10.	Are your DS able to support compliance requirements from carrier point of view?
11.	Which hurdles do you see for the carrier in order to achieve a fully connected E2E transport chain?
12.	Which requirements must be fulfilled in your opinion, in order to realize a fully connected E2E transport chain?
13.	Does a central cloud-based platform able to shape a data-driven value-added process in real-time for the carrier along the transport chain?
14.	To which extent the quality of transport service by the carrier could be reduced (or increased) with the assistance of a central cloud-based platform?
D. Concluding remarks	
15.	Do you see other opportunities or risks for the provider of DS along the transport chain in the commercial road haulage?
16.	Which aspects are most important for you we did not mention yet?

## Appendix 2

**Table 6:** Coding examples (focusing characteristics and typologies of digital platforms)

Transcript data from interview	Interview organization	Line-by-line coding	Conceptualization / Categorization	Element / Platform Type	DP Research Focus
We can return data via our platform and improve the order to payment process between shippers and carriers.	CARGOCLOUD	Improves payment service, links shippers and carriers	Optimization service, platform boundary	Order-to- Payment, Shipper-to- Carrier	Characteristics
To give an example here, we process our customers’ experience values at loading and unloading points accordingly, so that we can already calculate in advance how long an unloading or loading at customer X will take on average.	TRUCKAHEAD	Process historical transport delivery data for advanced analysis	Analytical service	Delivery Performance	Characteristics
We basically record the transport order, link it to the tracking system, and that’s the core service by now.	VISACARGO	Consolidates order data from tracking system	Data activity, data source	Data aggregation, tracking device	Characteristics
We offer our customers an open marketplace, a freight marketplace, where shippers can publish transport requests and transport service providers can then submit corresponding cargo space offers in order to receive the transport order.	DIGITALTRANS	Enables demand and supply exchange of freight based on matching resources	Freight resource handling	Digital freight exchange (DFE)	Typology
If the forwarder proceeds with the order, then it is already over here, because then we do not know whether the forwarder checks the license of the carrier, because according to 7C we are also liable for his subcontractors.	STEPFORWARD	Supports compliance checks for forwarders during subcontracting order	Transport process support	Transport compliance provider (TCP)	Typology
Hence, we are creating the connectivity platform with the option for our OEM subsidiaries or sister companies to offer their services, and we have enabled a scalable business, taking [company name anonymized] as an example (…).	ROADSHIPPER	Connects vehicle OEM as a single point to enable data services	Transport data connectivity	Transport system integrator (TSI)	Typology

## Footnotes

1 https://www.siliconroundabout.org.uk/2021/01/can-a-1tn-freight-forwarding-industry-be-disrupted-jeff-bezos-eric-schmidt-and-travis-kalanick-think-it-can-beacon-ceo-fraser-robinson-explains/ (Retrieved 28 May 2021)
